# Commuters’ Personal Exposure Assessment and Evaluation of Inhaled Dose to Different Atmospheric Pollutants

**DOI:** 10.3390/ijerph17103357

**Published:** 2020-05-12

**Authors:** Francesca Borghi, Andrea Spinazzè, Giacomo Fanti, Davide Campagnolo, Sabrina Rovelli, Marta Keller, Andrea Cattaneo, Domenico Maria Cavallo

**Affiliations:** Department of Science and High Technology, University of Insubria, 22100 Como, Italy; andrea.spinazze@uninsubria.it (A.S.); g.fanti@studenti.uninsubria.it (G.F.); davide.campagnolo@uninsubria.it (D.C.); sabrina.rovelli@uninsubria.it (S.R.); mkeller@studenti.uninsubria.it (M.K.); andrea.cattaneo@uninsubria.it (A.C.); domenico.cavallo@uninsubria.it (D.M.C.)

**Keywords:** air pollution, exposure assessment, inhaled dose, commuters, environments, outdoor

## Abstract

Several studies evaluating exposure to pollutants in microenvironments (MEs) are available in the scientific literature, but studies that evaluate the inhaled doses of pollutants are few in number. Therefore, this study aimed to evaluate the exposure of commuters to different pollutants (i.e., nitrogen dioxide [NO_2_] and fractionated particulate matter [PM], including ultrafine particles [UFPs]) using miniaturized and portable real-time monitoring instruments in selected MEs; the inhaled doses of these pollutants were estimated for each of these MEs. Measurements were performed along a typical commute, considering different traffic and nontraffic MEs. Experimental data were collected over four working weeks in two different seasons (winter and summer). Different portable and miniaturized instruments were used to evaluate PM and NO_2_ exposure. Furthermore, physiological parameters were evaluated using a heart rate monitor. The principal results show that higher exposure levels were measured in Underground (for all PM fractions and NO_2_) and in Car (UFP), while lower levels were measured in Car (PM and NO_2_) and in Train (UFP). In contrast, higher values of the inhaled cumulative dose were estimated in environments defined as Other, followed by Walking (ht), while lower values were observed in Walking (lt) and in Car.

## 1. Introduction

It is well known that people are continuously exposed to different environmental factors, such as air pollutants. For this reason, it is necessary to evaluate the human exposure (defined as the contacts of a chemical, physical, or biological agent with the outer boundary of an organism [1]) to these pollutants. These chemical factors may cause serious health problems, and their adverse effects are particularly critical in urban areas, representing hotspots, specifically due to traffic emissions [2]. Moreover, travel microenvironments (MEs) contain high levels of different air pollutants [2]. Although the time spent commuting represents only a small fraction of the day, this activity may significantly contribute to both overall exposure to and the inhalation of pollutants [2]. Different studies have been conducted to evaluate the exposure of commuters to pollutants across Europe, considering different traffic (and nontraffic) MEs, as reported in the literature [3,4], and to assess variations among commuting modes. As reported in a recent paper [2], the number of studies based on personal monitoring during a simulated daily commute, conducted in different cities, has increased in the last few years, considering different transport modes [5,6,7,8,9,10,11,12,13,14,15,16,17,18]. For example, a recent paper reported that commuting via motorized modes leads to higher exposure to some pollutants (such as PM_2.5_ and black carbon) than commuting via pedestrian networks or cycling. However, other studies identified higher exposure (for the same pollutants) during cycling [2]. In summary, consensus regarding the ‘cleanest’ or ‘dirtiest’ commuting route has not been reached, although authors agree on the fact that ambient or background air quality monitoring does not accurately reflect the variability of pollutant exposure concentrations [2].

Furthermore, to better understand the determinants of exposure levels in traffic MEs, it is important to note that most of the literature only assesses commuter exposures to airborne contaminants, but not the corresponding inhaled doses. The inhaled dose of airborne pollutants is determined based on the subject’s pulmonary ventilation rate and physical activity (other than on the subject’s exposure level to the airborne pollutant), but these parameters are often not considered in the experimental design of exposure assessment studies (generally due to technical issues associated with the measurement of some physiological parameters) [19]. However, assessments of inhaled doses of pollutants can be of significant interest for risk assessment, specifically in the case of commuters, because higher inhaled doses can be observed due to the high pollutant exposure which is typically associated with urban transit/traffic environments [20,21], and higher inhalation rates can be noted during active transport modes such as walking and cycling (i.e., increased physical effort leads to increased inhalation rates, and therefore, higher inhaled doses and lung deposition of pollutants) [19].

Therefore, this study aimed (i) to assess the exposure levels to different airborne pollutants, such as size-fractionated particulate matter (PM) (PM_1_, PM_2.5_, PM_4_, PM_10_, and total suspended particles [TSPs]), including ultrafine particles (UFPs) and nitrogen dioxide (NO_2_) measured across a commuting route, from a provincial to a big city in the northern region of Italy; (ii) to describe pollutant exposure levels across eight different MEs; (iii) to estimate the dose of the considered airborne pollutants inhaled during daily commuting and across different MEs, considering the subject’s physiological parameters; and (iv) to assess whether different inhaled doses, compared to external exposure, are observed in different MEs.

## 2. Materials and Methods

### 2.1. Study Design and Instrumentation

To simulate a typical home-to-work (and return) commuter route, a fixed route (for a total of 90 km) was defined a priori from a provincial city (‘home’ [Villa Guardia], 45°47′ N 9°01′ E) to an office located in Milan (‘Workplace’, 45°27′ N 9°11′ E), the largest city in Lombardy, Italy (Figure S1).

As reported by Longhin and collaborators [22], Milan is the biggest city in the Po Valley area, Italy, with 1.3 million inhabitants, and one of the most populous metropolitan cities in Europe, with more than 7.5 million inhabitants (considering the Milanese Metropolitan area). As reported elsewhere, [23], the Po Valley has experienced several air pollution episodes in the past decades, due to the (i) topography, (ii) high population density, and (iii) high atmospheric stability during wintertime. In particular, the main summer source of PM is vehicular traffic, while biomass burning emissions combined with local meteorological conditions contribute to the increased PM concentrations in winter.

With the use of a commuting route, various MEs that are typically visited by commuters were considered. Details regarding the MEs considered in this study are presented in Table S1, but in general, the MEs visited by the commuter were as follows: Walking (low traffic [lt] condition), Walking (high traffic [ht] condition), Bike, Car, Underground, Train, Indoor, and Other MEs (defined as the transition period [2 min] from on environment to another). Experimental data were collected over two working weeks (Monday to Friday) in two different seasons (winter campaign, 11 March, 2019–15 March, 2019 and 18 March, 2019–22 March, 2019; summer campaign, 8 July, 2019–12 July, 2019, 15 July, 2019–19 July, 2019; the monitoring on Thursday [11 July 2019] was cancelled due to a public transport strike and was rescheduled to the next available Thursday [25 July 2019]) to characterize weekly and seasonal pollutant concentration variability.

Portable and miniaturized monitors were used to assess exposure levels to different airborne pollutants. All the instruments were worn by one of the authors (F.G.) using a backpack. All instrument inlets were placed in the breathing zone of the operator, with 30 cm-radius hemispheres extending in front of the face (Figure S2). All instruments were checked daily, and all guidelines provided by the manufacturer were followed to ensure quality-controlled data. Instruments were also constantly checked during the monitoring phase to prevent instrument failure. All instruments were set up with an acquisition rate equal to 60 s.

Different portable instruments, both direct-reading and filter-based, were used to evaluate size-fractionated PM exposure. UFP exposure levels were measured using a portable diffusion size classifier (DiSCmini [DSC], Matter Aerosol AG, Wohlen AG, Swiss). The DSC used in this study can measure the concentration and the average size of the particles in the range of 10 < Dp < 700 nm. The continuous determination of size-fractionated PM concentrations was also performed using a second portable direct-reading monitor (Aerocet 831-Met One Instrument Inc., Grant Pass, Oregon, USA), which provides concentration data of the different PM fractions (PM_1_, PM_2.5_, PM_4_, PM_10_, and TSP). Finally, a miniaturized monitor was used for the evaluation of PM_2.5_ concentration (AirBeam [AB], HabitatMap Inc., Brooklyn, NY, USA). This monitor is based on an Arduino board, and it can detect particles in a range from 0.5 to 2.5 µm and a PM_2.5_ concentration up to 400 µg/m^3^. PM_2.5_ samples were collected using a GK2.05 sampler (BGI Inc., Waltham, MA, USA), operated with a sampling pump with a flow rate equal to 4 L/min; particles were collected using polytetrafluoroethylene filters. Mass concentration was determined by performing gravimetric analysis following a standard reference method [24,25]. The weighing procedure [26,27,28] considered the conditioning of filters in a controlled environment (temperature [T], 20 ± 1 °C; relative humidity [RH], 50 ± 5%) for a minimum of 24 h. Subsequently, the filters were weighted before and after sampling using a microbalance (Gibertini Micro 1000, Novate, Milan, Italy). Gravimetric data were used to correct PM data outcomes from direct-reading instruments, providing a daily correction factor, applied a posteriori to the whole PM dataset.

The measurement of NO_2_ concentrations was performed using a miniaturized electrochemical monitor (CairClip NO_2_, Cairpol; La Roche Blanche, France). The subject’s heart rate was measured using a heart rate monitor (Suunto 9). This instrument was also used to acquire Global Positioning System data, with the same acquisition rate as that of the other instruments (i.e., 60 s).

### 2.2. Statistical Analysis and Inhaled Dose Estimation

Following the well-established practices in statistics and the literature, data obtained using direct-reading instruments were examined and handled to exclude zero and unreliable data. For this reason, concentration distributions were truncated above the 99th percentile and below the 1st [29]. Moreover, following the literature [30] on the validation and evaluation of microsensors, NO_2_ values below the calculated limit of detection (LOD) (‘LOD’ = 1.692 µg/m^3^) were replaced with LOD/2, which was relatively justified [31]. Furthermore, following the technical references of direct-reading instruments, PM data obtained in extreme microclimatic conditions (RH > 80%; T > 50 °C) were eliminated to exclude data which was subject to recognized environmental interference. As mentioned, the error associated with the PM direct-reading instruments was managed using a calculated correction factor. The correction factor, calculated by dividing daily PM concentrations measured gravimetrically with the daily average PM concentration measured simultaneously using direct-reading instruments, was applied to data measured from direct-reading instrument monitoring [27,32]. UFP mass concentrations were calculated based on the number concentrations, particle diameter, and mean mass density factors [21].

In this paper, a descriptive statistic (reporting number of observations, mean, minimum, maximum, and standard deviation) was performed on the total dataset and for each kind of ME considered in this study to provide an overview of the data obtained during the two monitoring periods. Moreover, to comprehensively evaluate the exposure concentrations in each ME, the average values (the total values for different seasons) measured in different environments were reported.

Descriptive statistics regarding the heart rate (beats per minute [bpm]) measured in each ME and the calculated ventilation rate (L/min) were also reported. As reported in the literature [2], the pollutant inhaled dose can be estimated as the product of the measured exposure concentration, the ventilation rate, and the time spent in each specific ME. In this regard, the subject’s ventilation rate was calculated following methods described in the literature [33], where the ventilation rate (L/min) was calculated as reported in Equation (1), considering the heart rate (bpm) of the subject. The descriptive statistic of the inhaled dose was reported in this study as the average dose calculated in each ME:

VE = 0.00071 × HR2.17(1)

Equation (1) Calculation of the ventilation rate [33]. VE: ventilation rate (L/min); HR: heart rate (bpm).

To assess the distribution of exposure data, a Kolmogorov-Smirnov test was performed. Once the distributions were verified to be neither normally nor log-normally distributed, a nonparametric test (Mann-Whitney U test) was performed to evaluate the differences of pollutant exposure levels among MEs. Data were analyzed using the Statistical Package for the Social Sciences Statistic version 20.0 (IBM, Armonk, NY, USA), and a significance level of 0.05 was used in all statistical tests.

## 3. Results and Discussions

### 3.1. Descriptive Analysis: Pollutant Exposure Levels and Physiological Parameters

During the two monitoring periods, an evaluation of pollutant exposure levels was performed across different MEs. Table 1 reports descriptive statistics regarding the total and seasonal (winter and summer) levels of exposure, while Figure S3 represents the average contribution of differential PM fractions to the total values (for the whole period and for the two considered seasons).

As expected, during the summer period, the average exposure to airborne pollutants was lower than that measured during the winter, except for UFP and NO_2_.

Figure S3 shows the contribution of the different fractions on the total dataset: PM_1_ represents 40% of the total dataset during the summer period and 30% during the winter period. PM_1–2.5_ and PM_2.5–4_ were similar during the two periods considered (12% and 10%, respectively), like PM_>10_ (equal to 15%). The PM_4–10_ fraction was more significant during the winter period (31% of the total dataset) than in the summer period (25% of the total dataset).

To evaluate the changes and variations of exposure levels as a function of the considered MEs, descriptive statistics of the average exposure levels found across MEs are reported in Table 2. The MEs considered in this study were the following: Walking (lt), Walking (ht), Bike, Car, Underground, Train, Indoor, and Other MEs. Higher exposure levels were observed in Underground (for all PM fractions and NO_2_) and in Car (UFP), while lower exposure levels were observed in Car (PM and NO_2_) and in Train (UFP). This trend was observed in the total database and in the seasonal datasets (summer and winter). Higher summer exposure concentrations were observed in specific MEs: exposure to UFP was higher during summer than during winter in Walking (lt), Car, Train, Indoor, and Other MEs, while PM_1_ summer exposure was higher in Walking (lt) and in the Indoor MEs. NO_2_ exposure levels were also higher in the warm season in Walking (lt), Bike, Car, and Other MEs. Finally, higher concentrations during the summer were observed in Walking (lt) for PM_2.5_, PM_4_, and PM_10_. Generally, greater differences between summer and winter exposure concentrations were measured in the Underground environment (ranging from 0.5 µg/m^3^ UFP to 80.5 µg/m^3^ TSP), while lower differences were observed in the Train environment (ranging from 0.7 µg/m^3^ UFP to 5.6 µg/m^3^ TSP).

As presented in Figure S4, it is possible to observe the trend of the differential concentrations calculated in the different MEs (calculated for the total and seasonal databases). PM_1_ makes an important contribution to the total database in the Car ME during the winter period (59%), while it has similar percentages to those found in other MEs during the summer period (51%). In fact, during winter, the percentages of PM_1_ observed in the other MEs ranged from 29% (Walking [lt]) to 37% (Train), while they varied from 30% (Underground) to 46% (Train) during summer. The PM_1–2.5_ fraction was considered to have a percentage contribution that was significantly similar in the different MEs and during both seasons. The percentages varied from 7% (Train) to 15% (Underground) during winter and from 5% (Train) to 15% (Underground) during summer. The same trend was observed in the PM_2.5–4_ fraction, which varied from 8% (Train and Walking [lt]) to 14% (Underground) during winter and from 6% (Train) to 13% (Underground) during summer. The PM_4–10_ fraction was shown to have a lesser influence on the total database in the Car environment during summer (16%) and winter (14%) compared to the other MEs. In fact, the percentages of this fraction in the other MEs ranged from 25% (Train) to 40% (Walking [lt]) during winter and from 21% (Train) to 32% (Walking (lt)) during summer. Finally, the PM_>10_ fraction was similar in the different MEs and in the different seasons considered, except for the Train (winter, 24%; summer, 22%) and Indoor environments during the winter period (19%). In fact, the percentages ranged from 9% (Car) to 16% (Walking [lt]) during winter and from 10% (Walking [lt], Walking [ht], Bike) to 15% (Underground and Other MEs) during summer

In addition to exposure data, the subject’s physiological parameters (heart rate) were obtained during the whole monitoring campaign and used to calculate the ventilation rate [2]. In Table S2, a descriptive statistic regarding the heart rate and ventilation rate calculated for each ME is reported. As expected, higher values were observed in active commuting (101–104 bpm for Walking and Cycling), while lower values, equal to 66 and 69 bpm, were observed in Car and Train MEs. Hence, ventilation rate was higher in active transport modes than in passive transport modes.

### 3.2. Inhaled Dose Across Different Microenvironments (MEs) and Differences in Exposure Levels Across MEs

A descriptive statistic of the inhaled dose estimated for each pollutant following Equation (2) (where the inhaled dose is the mass of PM that enters the subject respiratory system, Conc. is the PM concentration, T is the time spent commuting and VE is the minute pulmonary ventilation rate of the subject) is reported in Table 3:

Inhaled Dose: Conc. × T × VE(2)

Equation (2) Inhaled dose (µg) estimation. Conc: exposure concentration (µg/m^3^); T: time (min); VE: pulmonary ventilation rate (m^3^/min).

In general, higher values of inhaled doses were estimated in environments defined as Other MEs, followed by Walking (ht), while lower values were observed in Walking (lt) and Car.

The trend reported above indicates the general trend of inhaled doses according to the ME, but it is important to emphasize that this may vary according to the fraction of the particulate and to the pollutant considered. For this reason, in Table 4, the inhaled dose values are reported according to the ME considered.

The average inhaled dose values are always higher in the environment defined as ‘Other’, probably since this ME is considered as a moment of transition from one environment to another, and is therefore influenced by a high variability (in terms of exposure concentrations and of VE). Moreover, during this period, the subject had to move quickly, in most cases changing transport modes. For this reason, it is likely that the exposure concentrations could have been influenced by the subject’s sudden movements. High inhaled dose values were also observed in Walking (ht) and Underground (and Indoor, only for UFP). It should be noted that although the exposures measured in the Walking (ht) environment were approximately one third of those measured in the Underground, and the time spent within that environment was approximately half that of either Walking (ht) (10 min on average) or Underground (24 min), the inhaled dose values estimated for these two MEs were in the same order of magnitude for all pollutants (with the exception of UFPs). Furthermore, the pulmonary ventilation rate measured in the Walking (ht) ME was among the highest observed ME during the whole monitoring period (18 L/min); this may explain the high inhaled doses estimated in this environment. Doses in the same order of magnitude were measured in the following environments: Bike, Train, and Indoor (except for UFP). Lower doses were estimated in Walking (lt) and Car (for all pollutants except UFP). The low values observed in the Car environment can be explained by the fact that in this ME, the lowest concentrations and pulmonary ventilation rate (7 L/min) were observed. The difference between the doses estimated for the two Walking environments (lt and ht) can be explained by the fact that in the Walking (lt) environment, the average time spent was approximately a quarter of that spent in the Walking (ht) environment (10 and 45 min, respectively). The other two parameters certainly appear to be significantly similar: the ventilation was equal to 18 and 19 L/min for Walking (lt) and Walking (ht), respectively. The measured concentrations of exposure were instead found to be similar for all pollutants (average differences between the two environments: 0.2 µg/m^3^, ranging from −6.2 to 4.4 µg/m^3^).

The obtained PM_2.5_ inhaled dose is different than that observed by Tan and collaborators [2]. In their study, the authors found that the highest mean inhaled dose was obtained for the Walking mode (23.1 µg), followed by Taxi (2.4 µg), Bus (3.0 µg), and Underground (2.6 µg). Regarding the ratios calculated between the inhaled dose values and exposure levels, lower ratios, i.e., close to 0, were observed in Walking (lt) and Car environments (ratio, 0:2), followed by Bike and Underground (ratio, 0:3), Train (ratio. 0:6), Indoor (ratio, 0:8), and Walking (ht) (ratio, 0:9). Ratio >1 was only observed in Other MEs (ratio, 1:2).

Moreover, once the exposure data were verified to be neither normally nor log-normally distributed (via Kolmogorov-Smirnov test, *p* < 0.001 for all pollutants), a nonparametric test (Mann-Whitney U test) was performed to evaluate the presence of statistically significant differences of pollutant exposure levels among different MEs. A summary of the results is reported in Table S3

In general, as reported in Table S3, measured exposure levels were found to be statistically different in different MEs, with some exceptions. For example, statistically significant differences in exposure levels were not observed for UFP exposure levels for Walking (lt) vs Indoor, Walking (ht) vs Bike, Walking (ht) vs Car, Bike vs Car, Bike vs Underground, and Car vs Underground. Statistically significant differences in exposure levels were not observed for PM_1_ and PM_2.5_ (measured via AB) for Indoor vs Train. Interestingly, statistically significant differences were not observed for exposure to NO_2_ or to all PM fractions (from PM_1_ to TSP) between Walking (ht) and Walking (lt), Walking (lt) and Other MEs, and Walking (ht) and Other MEs.

## 4. Conclusions

This study aimed to evaluate the exposure levels to different pollutants (NO_2_ and size-fractionated PM) in different MEs (traffic and nontraffic related) typically visited by commuters. To this end, different portable and miniaturized, direct-reading instruments were used for the measurement of airborne pollutant exposure; hence, the data were characterized by high temporal resolution (1-min acquisition rate).

The study’s design also included the use of a heart rate monitor, which allowed us to acquire real-time physiological data (heart rate). This monitor was subsequently used to calculate the pulmonary ventilation data required to estimate the inhaled dose of pollutants in each investigated ME. To date, probably due to technical–logistic issues related to the real-time measurement of physiological parameters (heart rate or ventilation rate), studies reporting data on the inhaled dose of pollutants, specifically across different traffic MEs, are still limited. Hence, this study may contribute to broadening the knowledge of this topic in the scientific literature.

For both the exposure assessment and the inhaled dose estimation in traffic environments, the results derived from available studies were not consistent with each other. This was probably caused by the different conditions observed in different commuting MEs, which made it difficult to obtain a consistent result between the different studies. Furthermore, regarding the estimation of the inhaled dose, other parameters were taken into consideration in addition to the pollutant exposure concentrations. Pulmonary ventilation rate and time spent in each ME can vary significantly from study to study and (specifically regarding the pulmonary ventilation rate) from subject to subject. Further studies regarding the evaluation of the inhaled dose of pollutants should be conducted in the future to standardize the conditions that led to the estimations of inhaled doses in certain MEs, and to assess which environments (and the boundary conditions [pollutant exposure concentrations, pulmonary ventilation rate, and time spent in a given ME]) are effective when determining the pollutant inhaled dose.

### 4.1. Strengths and Limitations

This study has the following strengths: several instruments were simultaneously used for the personal exposure assessment. Moreover, considering the design of the study, it was possible to identify and assess the exposure levels (and consequently, the values of the subject’s estimated inhaled dose) in a number of (traffic and nontraffic) MEs. Moreover, the route chosen for this study was defined a priori and always travelled by the same operator: in this way, a certain level of reproducibility was ensured. Again, due to the experimental design, (i) different versions of the same route, even (ii) on different days and (iii) in different seasons (summer and winter), were evaluated. Finally, regarding the calculation of the inhaled dose of pollutants, unlike most studies in the literature, the subject’s physiological parameters (heart rate, that allowed us to calculate the pulmonary ventilation rate) were obtained at a personal level, and tabular standard data were not used.

The study has the following limitations. The study was conducted on one single route (towards Milan). Moreover, due to the experimental design, different traffic conditions (high and low traffic) were assessed only while walking. In the case of the bicycle trip, only high traffic conditions were evaluated. Another limit of the study is that these results cannot be extended to other urban areas. In fact, the concentrations of pollutants measured in different MEs and the estimation of the inhaled dose are intrinsically characterized by a high variability. Furthermore, all the typical patterns of commuting in the city of Milan were not analyzed in this study, but an attempt was made to analyze the expected route of a typical commuter. In this regard, it is worth noting that more than 1 million people routinely commute on weekdays to the city of Milan for work or study (of this, 650,000 persons are residents of the city while 475,000 enter from other areas) [34]. Finally, it is necessary to recognize that two equations (Equations 1 and 2) were used to obtain data regarding the ventilation rate and the estimated inhaled dose; in this way, considering the use of different levels of approximation, it is necessary to consider the presence of an intrinsic error associated with the estimates.

### 4.2. Further Developments

Future developments should be assessed. First, in this study, the pulmonary ventilation rate was derived starting from the value (per minute) of the subject’s heart rate and then calculating the corresponding pulmonary ventilation rate. For this reason, it would be beneficial to determine whether the equation used for this calculation is applicable to larger populations and if the other equations present in the scientific literature provide different results. Moreover, since the estimation of the inhaled dose of pollutants was performed dependent on a simple equation (which is associated with pollutant exposure concentration, with the subject’s pulmonary ventilation rate, and with the time spent by the subject in a particular environment), it would be interesting to reanalyze the data related to the inhaled dose with a more detailed model. The mathematical multiple-path particle dosimetry model can be used, for example, as it is able to process data referring to the deposition of a determined PM fraction within the respiratory tract.

Finally, although already developed in another study [35], a sensitivity analysis will be performed using the data obtained from this study to evaluate which of the parameters included in the estimation of the inhaled dose (exposure concentration, pulmonary ventilation, and time spent in a particular environment) has the most significant influence on the inhaled dose.

## Figures and Tables

**Table 1:** Descriptive statistic performed on the total dataset and for the winter and summer campaigns (N: Number of Data; Min.: Minimum; Max.: Maximum; S.D.: Standard Deviation; LDSA: ung Deposited Surface Area; PM_2.5_ (AB): PM_2.5_ Measured via AirBeam). Data are reported as µgm^3^ (* article/cm^3^; ** nm; *** µm^2^/cm^3^). Data in italics refer to those used for the calculation of UFP mass.

Parameter	N	Min.	Max.	Mean	S.D.	Monitoring Period
UFP number *	8179	212	74436	9640	7027	Total
UFP diameter **	8228	<LOD	300.0	49.2	15.2	
UFP LDSA ***	8228	0.6	203.9	24.4	15.9	
UFP mass	8239	<LOD	197.3	3.7	4.1	
PM_1_	8365	0.1	174.8	10.2	12.5	
PM_1–2.5_	8026	<LOD	106.4	3.2	5.8	
PM_2.5_	8342	0.2	160.8	13.1	15.4	
PM_2.5–4_	8046	<LOD	139.9	3.4	5.9	
PM_2.5_ (AB)	7394	1.4	134.9	35.5	22.6	
PM_4_	8348	0.3	189.0	16.2	18.9	
PM_4–10_	8023	<LOD	303.5	8.3	13.2	
PM_10_	8345	0.6	378.5	24.0	28.4	
PM_>10_	8033	<LOD	399.6	4.5	9.1	
TSP	8340	0.6	480.6	28.2	33.0	
NO_2_	8690	0.9	478.5	30.5	52.7	
UFP number *	4014	477	63678	10133	7449	Winter
UFP diameter **	4063	<LOD	130.3	44.5	11.0	
UFP LDSA ***	4063	0.6	203.9	24.3	17.6	
UFP mass	4074	<LOD	197.3	3.2	4.5	
PM_1_	4164	0.3	174.8	11.1	13.8	
PM_1–2.5_	3744	<LOD	76.5	4.4	7.1	
PM_2.5_	4162	0.7	160.8	14.8	17.0	
PM_2.5–4_	3747	<LOD	92.2	4.5	6.7	
PM_2.5_ (AB)	3763	26.5	116.5	50.6	14.7	
PM_4_	4162	1.1	189.0	18.7	21.0	
PM_4–10_	3747	<LOD	303.5	11.3	16.6	
PM_10_	4162	1.1	378.5	28.6	33.0	
PM_>10_	3747	<LOD	399.6	5.7	11.0	
TSP	4162	1.5	480.6	33.5	38.6	
NO_2_	4389	0.9	478.5	29.0	50.6	
UFP number *	4165	212	74436	9164	6560	Summer
UFP diameter **	4165	18.7	300.0	53.8	17.2	
UFP LDSA ***	4165	1.8	168.0	24.4	14.0	
UFP mass	4165	0.1	73.9	4.2	3.5	
PM_1_	4201	0.1	70.2	9.2	11.0	
PM_1–2.5_	4282	<LOD	106.4	2.2	4.2	
PM_2.5_	4180	0.2	106.4	11.4	13.5	
PM_2.5–4_	4299	<LOD	139.9	2.4	4.8	
PM_2.5_ (AB)	3631	1.4	134.9	19.9	18.3	
PM_4_	4186	0.3	139.9	13.7	16.3	
PM_4–10_	4276	<LOD	183.1	5.8	8.4	
PM_10_	4183	0.6	190.6	19.5	22.1	
PM_>10_	4286	<LOD	214.9	3.5	7.0	
TSP	4178	0.6	223.8	22.9	25.1	
NO_2_	4301	0.9	478.5	32.0	54.7	

**Table 2:** Descriptive statistics (mean) of various microenvironments performed on the total dataset and for the winter and summer campaigns. Data are presented as µg/m^3^ (* particle/cm^3^; ** nm; *** LDSA: lung deposited surface area, µm^2^/cm^3^; PM_2.5_ (AB): PM_2.5_ measured via AirBeam). Data in italics refer to those used for the calculation of ultrafine particle mass.

	**Walking (lt)**			**Walking (ht)**			**Bike**			**Car**		
	**Total (Mean)**	**Winter (Mean)**	**Summer (Mean)**	**Total (Mean)**	**Winter (Mean)**	**Summer (Mean)**	**Total (Mean)**	**Winter (Mean)**	**Summer (Mean)**	**Total (Mean)**	**Winter (Mean)**	**Summer (Mean)**
UFP number *	9218	9384	9053	13735	16432	11484	15655	17824	13700	13843	14161	13447
UFP diameter **	46.7	45.1	48.3	46.9	42.9	50.5	44.4	44.2	44.6	51.5	47.2	57.0
UFP LDSA^***^	22.9	22.6	23.2	34.0	38.2	30.2	37.1	42.6	32.2	37.1	36.8	37.6
UFP mass	3.3	3.3	3.3	4.5	4.3	4.8	4.6	5.4	3.9	6.3	5.6	7.3
PM_1_	12.8	11.3	14.4	12.3	13.1	11.5	15.0	16.4	13.8	5.8	6.8	4.4
PM_1–2.5_	2.7	3.2	2.3	2.9	3.8	2.1	4.1	5.1	3.1	1.0	1.2	0.9
PM_2.5_	15.5	14.5	16.7	15.2	16.9	13.6	19.1	21.5	16.9	6.8	8.0	5.3
PM_2.5–4_	3.1	3.0	3.2	3.8	4.9	2.7	5.5	7.2	4.0	0.9	1.0	0.8
PM_2.5_ (AB)	38.5	49.8	25.5	37.5	51.5	24.3	37.5	50.7	25.5	31.1	46.1	11.3
PM_4_	18.6	17.5	19.9	19.0	21.8	16.3	24.6	28.7	20.9	7.7	9.0	6.1
PM_4–10_	13.5	15.7	10.9	10.2	13.7	7.0	14.3	19.7	9.5	1.6	1.6	1.4
PM_10_	32.1	33.2	30.8	29.2	35.5	23.3	38.9	48.4	30.4	9.3	10.6	7.5
PM_>10_	5.0	6.2	3.6	3.5	4.3	2.7	4.4	5.5	3.3	1.1	1.0	1.1
TSP	37.1	39.4	34.4	32.7	39.8	26.0	43.3	53.9	33.7	10.4	11.6	8.6
NO_2_	32.3	25.5	39.9	38.5	39.5	37.5	44.6	30.6	57.5	10.8	5.9	17.0
	**Underground**			**Train**			**Indoor**			**Other**		
	**Total (mean)**	**Winter (mean)**	**Summer (mean)**	**Total (mean)**	**Winter (mean)**	**Summer (mean)**	**Total (mean)**	**Winter (mean)**	**Summer (mean)**	**Total (mean)**	**Winter (mean)**	**Summer (mean)**
UFP number *	11195	12638	9932	5925	5518	6291	8531	7712	9229	10038	11559	8802
UFP diameter **	49.8	48.2	51.2	51.4	43.2	58.8	49.1	47.1	50.8	50.7	45.8	54.7
UFP LDSA ***	30.1	33.1	27.5	14.9	12.4	17.1	22.2	20.0	24.0	25.6	28.0	23.7
UFP mass	4.5	4.8	4.3	2.6	1.5	3.5	3.4	3.0	3.8	3.9	3.7	4.1
PM_1_	27.9	42.7	17.5	7.1	7.5	6.8	7.5	7.2	7.7	12.5	15.4	10.1
PM_1–2.5_	14.2	21.4	8.7	1.1	1.4	0.7	1.7	2.2	1.4	3.8	4.8	3.0
PM_2.5_	42.1	64.1	26.2	8.2	8.9	7.5	9.2	9.4	9.1	16.3	20.2	13.1
PM_2.5–4_	12.7	19.8	7.7	1.2	1.6	0.9	2.1	2.3	1.7	3.9	4.9	3.0
PM_2.5_ (AB)	54.4	66.3	46.7	32	50.9	14.2	32.1	49.7	14.0	35.6	50.9	22.8
PM_4_	54.8	83.9	33.9	9.4	10.5	8.4	11.3	11.7	10.8	20.2	25.1	16.1
PM_4–10_	26.1	40.7	15.9	4.0	5.0	3.1	5.0	6.2	4.1	9.4	12.5	7.0
PM_10_	80.9	124.6	49.8	13.4	15.5	11.5	16.3	17.9	14.9	29.6	37.6	23.1
PM_>10_	11.2	14.5	8.8	4.1	4.9	3.3	3.2	4.2	2.2	4.5	5.2	4.0
TSP	92.1	139.1	58.6	17.5	20.4	14.8	19.5	22.1	17.1	34.1	42.8	27.1
NO_2_	66.3	69.4	63.4	11.9	12.1	11.7	29.1	33.4	2.2	41.1	36.5	45.1

**Table 3:** Descriptive of the inhaled dose (µg) or airborne pollutants, reported as an average for each microenvironment and as total database.

Pollutant	Walking (lt)	Walking (ht)	Bike	Car	Underground	Train	Indoor	Other	Total
UFP	0.6	3.8	1.3	1.5	1.4	1.7	2.2	4.9	17.4
PM_1_	2.3	10.5	4.3	1.3	8.7	4.5	4.8	15.6	52
PM_1–2.5_	0.5	2.5	1.2	0.3	4.4	0.7	1.1	4.7	15.4
PM_2.5_	2.8	13	5.5	1.6	13.1	5.2	5.9	20.3	67.4
AB_2.5_	6.9	32.1	10.8	7.2	17	20.4	20.7	44.4	159.5
PM_2.5–4_	0.5	3.2	1.6	0.2	4	0.8	1.4	4.9	16.6
PM_4_	3.3	16.2	7.1	1.8	17.1	6	7.3	25.2	84
PM_4–10_	2.5	8.8	4.1	0.3	8.1	2.5	3.2	11.7	41.2
PM_10_	5.8	25	11.2	2.1	25.2	8.5	10.5	36.9	125.2
PM_>10_	0.9	3	1.3	0.3	3.5	2.6	2.1	5.7	19.4
TSP	6.7	28	12.5	2.4	28.7	11.1	12.6	42.6	144.6
NO_2_	5.8	32.9	12.8	2.5	20.7	7.6	18.7	51.3	152.3

**Table 4:** Inhaled dose values (µg) reported from the lowest to the highest for different pollutants. Under.: Underground; lt: Walking lt; ht: Walking ht.

UFP	PM_1_	PM_1–2.5_	PM_2.5_	PM_2.5–4_	PM_4_	PM_4–10_	PM_10_	PM_>10_	TSP	NO_2_
Lt	Car	Car	Car	Car	Car	Car	Car	Car	Car	Car
0.6	1.3	0.3	1.6	0.2	1.8	0.3	2.1	0.3	2.4	2.5
Bike	Lt	Lt	Lt	Lt	Lt	Lt	Lt	Lt	Lt	Lt
1.3	2.3	0.5	2.8	0.5	3.3	2.5	5.8	0.9	6.7	5.8
Under.	Bike	Train	Train	Train	Train	Train	Train	Bike	Train	Train
1.4	4.3	0.7	5.2	0.8	6.0	2.5	8.5	1.3	11.1	7.6
Car	Train	Indoor	Bike	Indoor	Bike	Indoor	Indoor	Indoor	Bike	Bike
1.5	4.5	1.1	5.5	1.4	7.1	3.2	10.5	2.1	12.5	12.8
Train	Indoor	Bike	Indoor	Bike	Indoor	Bike	Bike	Train	Indoor	Indoor
1.7	4.8	1.2	5.9	1.6	7.3	4.1	11.2	2.6	12.6	18.7
Indoor	Under.	Ht	Ht	Ht	Ht	Under.	Ht	Ht	Ht	Under.
2.2	8.7	2.5	13.0	3.2	16.2	8.1	25.0	3	28.0	20.7
Ht	Ht	Under.	Under.	Under.	Under	Ht	Under.	Under.	Under.	Ht
3.8	10.5	4.4	13.1	4	17.1	8.8	25.2	3.5	28.7	32.0
Other	Other	Other	Other	Other	Other	Other	Other	Other	Other	Other
4.9	15.6	4.7	20.3	4.9	25.2	11.7	36.9	5.7	42.6	51.3

## Supplementary Materials

The following are available online at https://www.mdpi.com/1660-4601/17/10/3357/s1, Figure S1: Lombardy region (Italy). Red text presents the commuters’ route chosen for this study; Figure S2: Setup of the instruments placed in a backpack. Inlets were placed in the breathing zone of the operator; Figure S3: Descriptive statistic of the differential concentration calculated for the total dataset and for the seasonal datasets (summer and winter). Green: PM_1_; black: PM_1–2.5_; grey: PM_2.5–4_; white: PM_4–10_; light blue: PM_>10_; Figure S4: Differential concentration (µg/m^3^ and %) calculated for the different microenvironments (total and seasonal dataset). Green: PM_1_; black: PM_1–2.5_; grey: PM_2.5–4_; white: PM_4–10_; light blue: PM_>10_; Table S1: Summary of the microenvironments (MEs) considered in this study. Hour and time of stay refers to those a priori planned, even if small variations should be considered. (LT: low traffic condition; HT: high traffic condition; n.a.: not available). * Return trip—these MEs refer to the same MEs frequented during the first part of the journey; Table S2: Physiological parameters (heart rate and calculated ventilation rate) reported for the total and for microenvironment dataset (bpm: beats per minute); Table S3: Summary of the Mann-Whitney U test. Values represent the level of significance (*p*). Pair comparisons with *p* < 0.05 are reported in italics: Under.: Underground; lt: Walking lt; ht: Walking ht.
