# Supplementary material for: Commuters’ Personal Exposure Assessment and Evaluation of Inhaled Dose to Different Atmospheric Pollutants
Source: Int J Environ Res Public Health. 2020 May 12;17(10):3357. doi: 10.3390/ijerph17103357 (PMC7277859; doi:10.3390/ijerph17103357)
Supplement: Supplementary file 1 [file ijerph-17-03357-s001.pdf]

Commuter’s personal exposure assessment and evaluation of inhaled dose to different atmospheric pollutants

Francesca Borghi[1,*]; Andrea Spinazzè[1]; Giacomo Fanti[1]; Davide Campagnolo[1]; Sabrina Rovelli[1]; Marta Keller[1]; Andrea Cattaneo[1]; Domenico Maria Cavallo[1]

[1] Department of Science and High Technology, University of Insubria, Como, Italy; andrea.spinazze@uninsubria.it; g.fanti@studenti.uninsubria.it; davide.campagnolo@uninsubria.it; sabrina.rovelli@uninsubria.it; mkeller@studenti.uninsubria.it; andrea.cattaneo@uninsubria.it; domenico.cavallo@uninsubria.it.

* Correspondence: f.borghi2@uninsubria.it; +39-031-238-6645 (F.B)

Supplementary material

Figure S1. Lombardy region (Italy). Red text presents the commuters’ route chosen for this study.

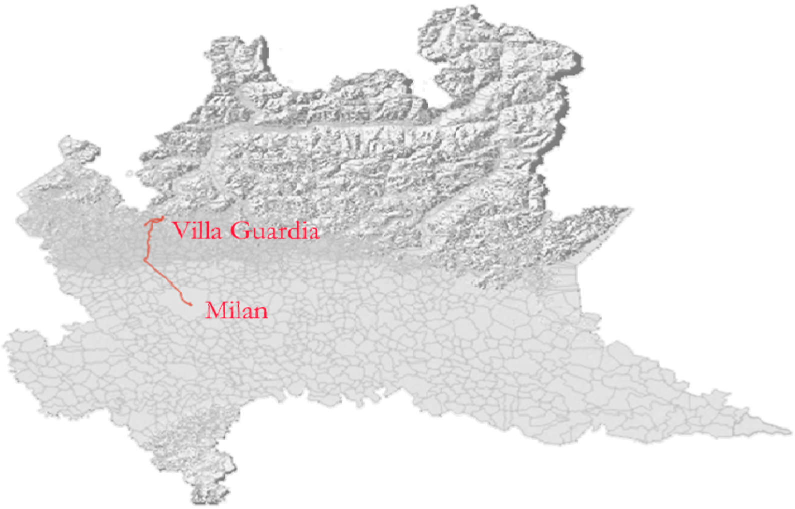

Figure S2. Setup of the instruments placed in a backpack. Inlets were placed in the breathing zone of the operator.

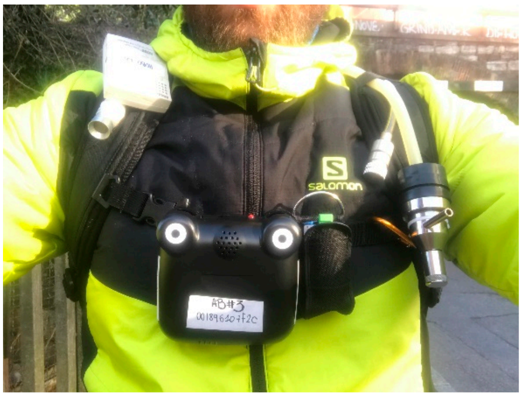

Figure S3. Descriptive statistic of the differential concentration calculated for the total dataset and for seasonal dataset (summer and winter). Green: PM_1; black: $PM_{1-2.5}$; grey: $PM_{2.5-4}$; white: PM_{4-10}; light blue: $PM_{>10}$.

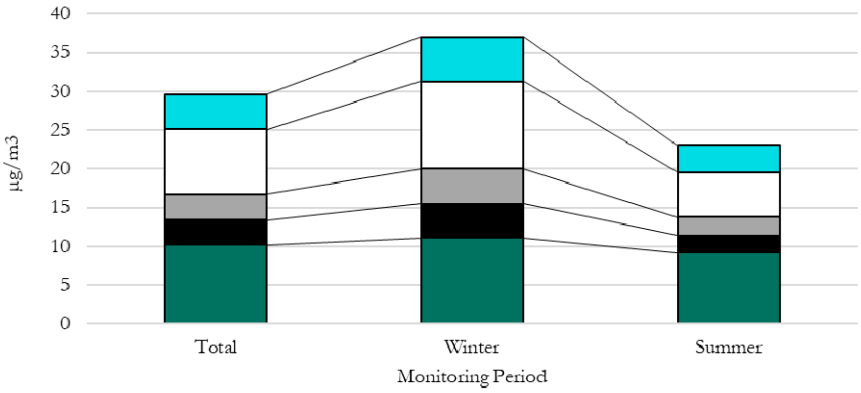

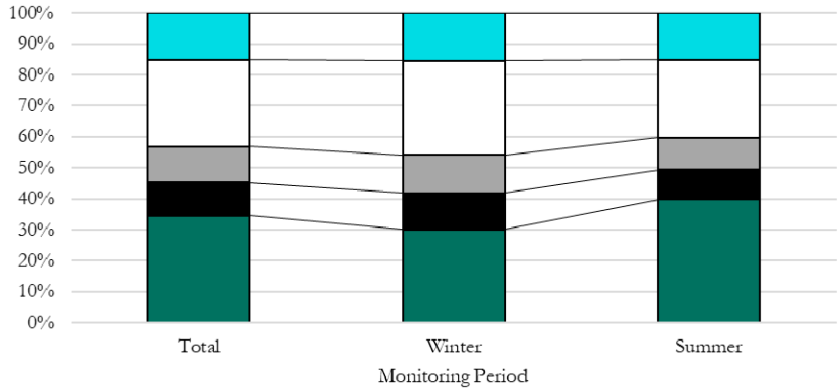

Figure S4. Differential concentration (μg/m³ and %) calculated for the different micro-environments (total and seasonal dataset). Green: PM_1; black: $PM_{1–2.5}$; grey: $PM_{2.5–4}$; white: $PM_{4–10}$; light blue: $PM_{>10}$.

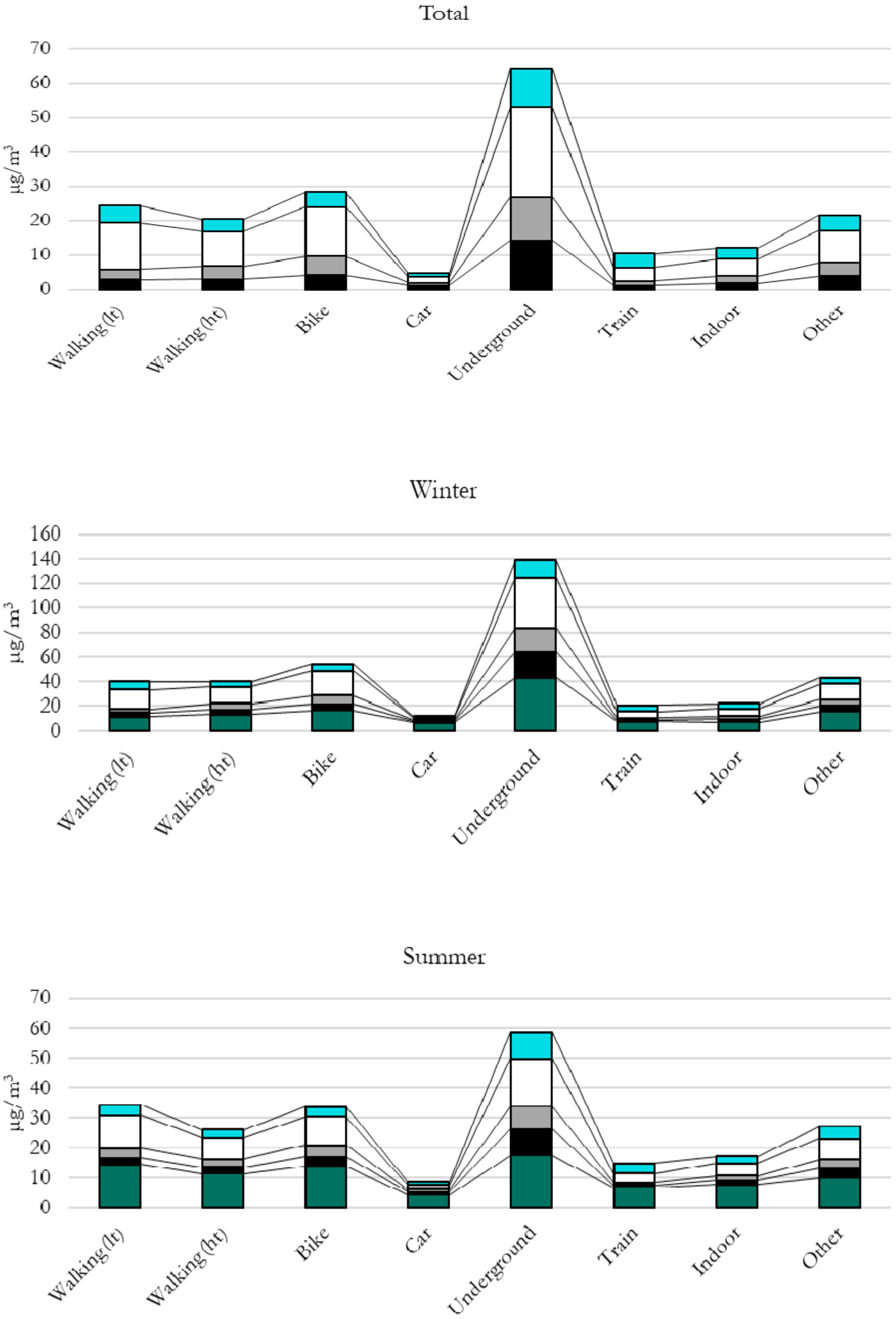

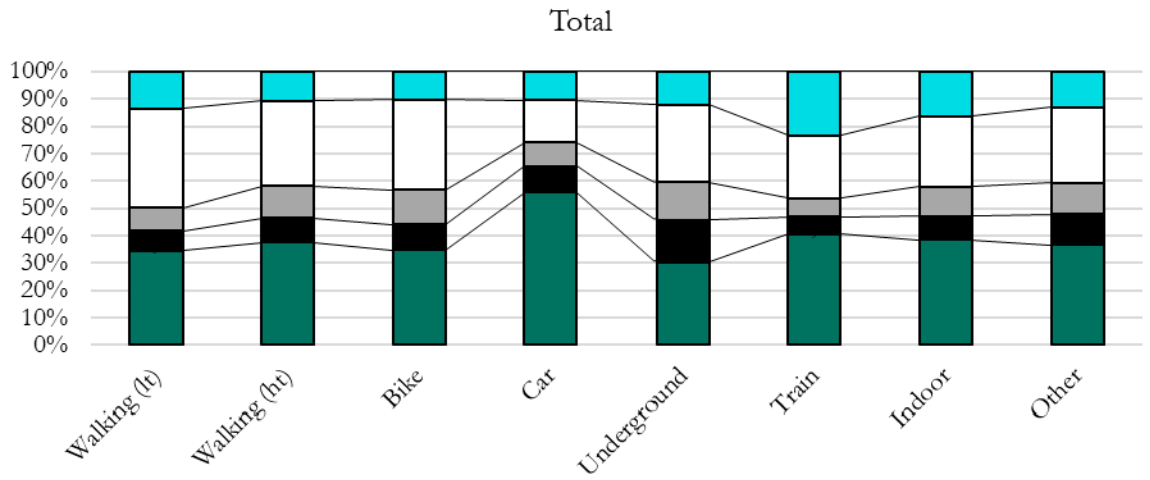
Total
100%
90%
80%
70%
60%
50%
40%
30%
20%
10%
0%
Walking (lt)
Walking (ht)
Bike
Car
Underground
Train
Indoor
Other

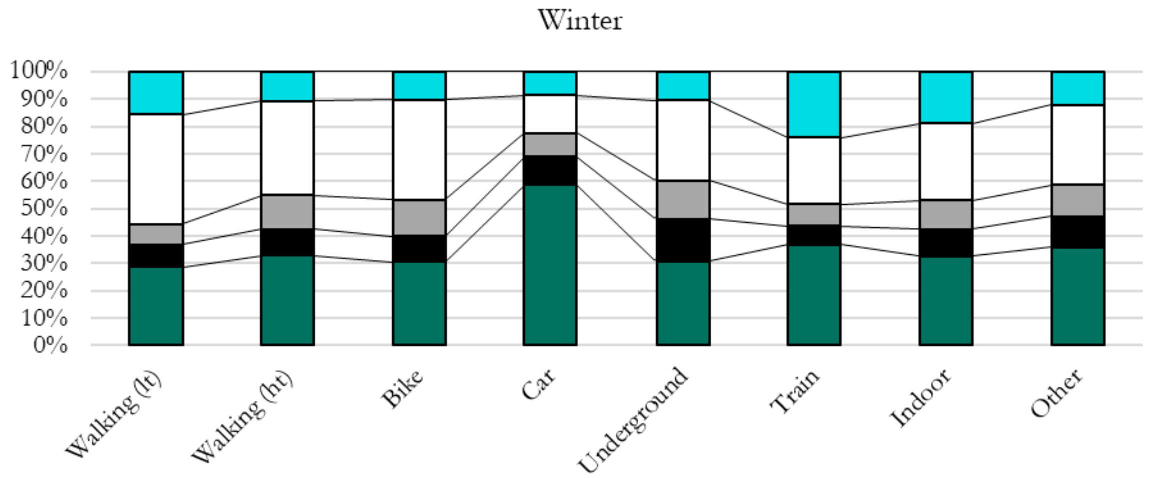
Winter
100%
90%
80%
70%
60%
50%
40%
30%
20%
10%
0%
Walking (lt)
Walking (ht)
Bike
Car
Underground
Train
Indoor
Other

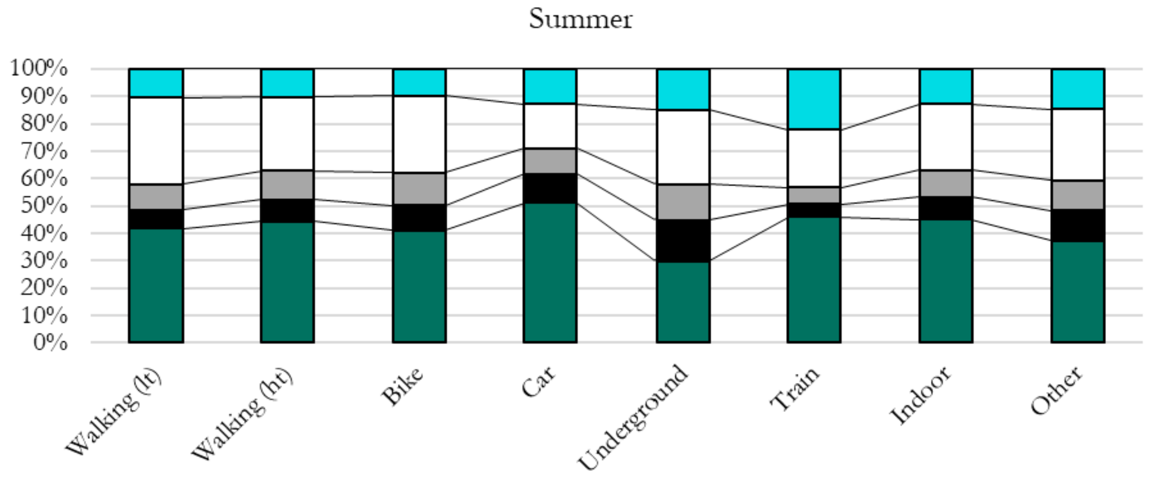
Summer
100%
90%
80%
70%
60%
50%
40%
30%
20%
10%
0%
Walking (lt)
Walking (ht)
Bike
Car
Underground
Train
Indoor
Other

Table S1. Summary of the micro-environments (MEs) considered in this study. Hour and time of stay refers to those a priori planned, even if small variations should be considered. (LT: low traffic condition; HT: high traffic condition; n.a.: not available). *Return trip – these MEs refer to the same MEs frequented during the first part of the journey.

ME	Hour (from–to; min)	Time of stay (min)	Route length (km)
Car	7:50–8:10	20	10
Walking (lt)	8:25–8:35	10	0.7
Train	8:45–9:35	50	45
Walking (lt)	9:35–9:55	20	1.5
Walking (ht)	9:55–10:05	10	0.5
Underground	10:05–10:15	10	2.5
Walking (ht)	10:20–10:30	10	0.6
Cycling	10:30–10:50	20	3
Indoor	10:50–12:00	70	n.a
Walking (ht)*	12:00–12:10	10	0.6
Underground*	12:10–12:20	10	2.5
Walking (ht)*	12:20–12:30	10	0.5
Walking (lt)*	12:30–12:50	20	1.5
Train*	13:20–14:10	50	45
Walking (lt)*	14:10–14:20	10	0.7
Car*	14:20–14:40	20	10

Table S2. Physiological parameters (heart rate and calculated ventilation rate) reported for the total and for micro-environment dataset (bpm: beats per minute).

Physiological parameters					
Environment	Min.	Max.	Mean	S.D.	
Total	46	209	81	26	Heart rate (bpm)
	3	77	11	9	Ventilation rate (l/min)
Walking (lt)	52	187	101	35	Heart rate (bpm)
	4	60	18	14	Ventilation rate (l/min)
Waking (ht)	49	194	104	35	Heart rate (bpm)
	3	65	19	14	Ventilation rate (l/min)
Bike	53	161	104	19	Heart rate (bpm)
	4	44	18	6	Ventilation rate (l/min)
Car	52	97	69	8	Heart rate (bpm)
	4	15	7	2	Ventilation rate (l/min)
Underground	47	190	88	25	Heart rate (bpm)
	3	63	13	10	Ventilation rate (l/min)
Train	46	191	66	11	Heart rate (bpm)
	3	63	7	4	Ventilation rate (l/min)
Indoor	46	165	77	17	Heart rate (bpm)
	3	46	9	5	Ventilation rate (l/min)
Other	48	209	85	27	Heart rate (bpm)
	3	77	12	10	Ventilation rate (l/min)

Table S3. Summary of the Mann-Whitney U test. Values represent the level of significance (p). Pair comparisons with $p<0.05$ are reported in italics: Under.: Underground; lt: Walking lt; ht: Walking ht.

Compared MEs		UFP	PM_{1}	$PM_{1–2.5}$	$PM_{2.5}$	$PM_{2.5–4}$	$PM_{2.5}$ (AB)	PM_{4}	$PM_{4–10}$	PM_{10}	$PM_{>10}$	TSP	NO_2
Lt	Ht	*<0.001*	0.129	0.211	0.171	0.203	0.663	0.182	0.115	0.440	0.211	0.270	0.393
Lt	Bike	*<0.001*	*<0.001*	*<0.001*	*<0.001*	*<0.001*	0.735	*<0.001*	*<0.001*	*<0.001*	*0.017*	*<0.001*	0.126
Lt	Car	*<0.001*	*<0.001*	*<0.001*	*<0.001*	*<0.001*	*<0.001*	*<0.001*	*<0.001*	*<0.001*	*<0.001*	*<0.001*	*<0.001*
Lt	Under.	*<0.001*	*<0.001*	*<0.001*	*<0.001*	*<0.001*	*<0.001*	*<0.001*	*<0.001*	*<0.001*	*<0.001*	*<0.001*	*<0.001*
Lt	Train	*<0.001*	*<0.001*	*<0.001*	*<0.001*	*<0.001*	*<0.001*	*<0.001*	*<0.001*	*<0.001*	0.847	*<0.001*	*<0.001*
Lt	Indoor	0.402	*<0.001*	*0.001*	*<0.001*	*<0.001*	*<0.001*	*<0.001*	*<0.001*	*<0.001*	*<0.001*	*<0.001*	*<0.001*
Lt	Other	*0.002*	0.381	0.236	0.313	0.826	*0.030*	0.429	*0.015*	0.221	0.118	0.419	*0.037*
Lt	Bike	0.577	*0.001*	*<0.001*	*<0.001*	*<0.001*	0.965	*<0.001*	*<0.001*	*<0.001*	*<0.001*	*<0.001*	*0.001*
Ht	Car	.154	*<0.001*	*<0.001*	*<0.001*	*<0.001*	*<0.001*	*<0.001*	*<0.001*	*<0.001*	*<0.001*	*<0.001*	*<0.001*
Ht	Under.	*0.011*	*<0.001*	*<0.001*	*<0.001*	*<0.001*	*<0.001*	*<0.001*	*<0.001*	*<0.001*	*<0.001*	*<0.001*	*<0.001*

Ht	Train	*<0.001*	*<0.001*	*<0.001*	*<0.001*	*<0.001*	*<0.001*	*<0.001*	*<0.001*	*<0.001*	*0.048*	*<0.001*	*<0.001*
Ht	Indoor	*<0.001*	*<0.001*	*<0.001*	*<0.001*	*<0.001*	*<0.001*	*<0.001*	*<0.001*	*<0.001*	*<0.001*	*<0.001*	*<0.001*
Ht	Other	*<0.001*	0.251	0.998	0.671	0.075	*0.003*	0.389	0.121	0.347	*<0.001*	0.538	0.053
Bike	Car	0.690	*<0.001*	*<0.001*	*<0.001*	*<0.001*	*<0.001*	*<0.001*	*<0.001*	*<0.001*	*<0.001*	*<0.001*	*<0.001*
Bike	Under.	0.085	*<0.001*	*<0.001*	*<0.001*	*<0.001*	*<0.001*	*<0.001*	*<0.001*	*<0.001*	*<0.001*	*<0.001*	*<0.001*
Bike	Train	*<0.001*	*<0.001*	*<0.001*	*<0.001*	*<0.001*	*<0.001*	*<0.001*	*<0.001*	*<0.001*	*<0.001*	*<0.001*	*<0.001*
Bike	Indoor	*<0.001*	*<0.001*	*<0.001*	*<0.001*	*<0.001*	*<0.001*	*<0.001*	*<0.001*	*<0.001*	*<0.001*	*<0.001*	*<0.001*
Bike	Other	*<0.001*	*<0.001*	*<0.001*	*<0.001*	*<0.001*	*0.021*	*<0.001*	*<0.001*	*<0.001*	0.124	*<0.001*	*<0.001*
Car	Under.	0.307	*<0.001*	*<0.001*	*<0.001*	*<0.001*	*<0.001*	*<0.001*	*<0.001*	*<0.001*	*<0.001*	*<0.001*	*<0.001*
Car	Train	*<0.001*	*0.002*	0.197	*0.005*	*0.001*	*0.008*	*0.001*	*<0.001*	*<0.001*	*<0.001*	*<0.001*	*<0.001*
Car	Indoor	*<0.001*	*<0.001*	*<0.001*	*<0.001*	*<0.001*	*0.027*	*<0.001*	*<0.001*	*<0.001*	*<0.001*	*<0.001*	*<0.001*
Car	Other	*<0.001*	*<0.001*	*<0.001*	*<0.001*	*<0.001*	*<0.001*	*<0.001*	*<0.001*	*<0.001*	*<0.001*	*<0.001*	*<0.001*
Under.	Train	*<0.001*	*<0.001*	*<0.001*	*<0.001*	*<0.001*	*<0.001*	*<0.001*	*<0.001*	*<0.001*	*<0.001*	*<0.001*	*<0.001*

Under.	Indoor	*<0.001*	*<0.001*	*<0.001*	*<0.001*	*<0.001*	*<0.001*	*<0.001*	*<0.001*	*<0.001*	*<0.001*	*<0.001*	*<0.001*
Under.	Other	*<0.001*	*<0.001*	*<0.001*	*<0.001*	*<0.001*	*<0.001*	*<0.001*	*<0.001*	*<0.001*	*<0.001*	*<0.001*	*<0.001*
Train	Indoor	*<0.001*	0.410	*<0.001*	*<0.001*	*<0.001*	0.250	*<0.001*	*<0.001*	*<0.001*	*<0.001*	*<0.001*	*<0.001*
Train	Other	*<0.001*	*<0.001*	*<0.001*	*<0.001*	*<0.001*	*<0.001*	*<0.001*	*<0.001*	*<0.001*	*<0.001*	*<0.001*	*<0.001*
Indoor	Other	*<0.001*	*<0.001*	*<0.001*	*<0.001*	*<0.001*	*<0.001*	*<0.001*	*<0.001*	*<0.001*	*<0.001*	*<0.001*	*0.001*
